# Eosinophil-Derived Osteopontin Induces the Expression of Pro-Inflammatory Mediators and Stimulates Extracellular Matrix Production in Nasal Fibroblasts: The Role of Osteopontin in Eosinophilic Chronic Rhinosinusitis

**DOI:** 10.3389/fimmu.2022.777928

**Published:** 2022-03-02

**Authors:** Hyun-Woo Yang, Joo-Hoo Park, Min-Sik Jo, Jae-Min Shin, Dae Woo Kim, Il-Ho Park

**Affiliations:** ^1^ Upper Airway Chronic Inflammatory Diseases Laboratory, Korea University College of Medicine, Seoul, South Korea; ^2^ Medical Device Usability Test Center, Guro Hospital, Korea University College of Medicine, Seoul, South Korea; ^3^ Department of Otorhinolaryngology-Head & Neck Surgery, Boramae Medical Center, Seoul National University College of Medicine, Seoul, South Korea; ^4^ Department of Otorhinolaryngology-Head and Neck Surgery, Korea University College of Medicine, Seoul, South Korea

**Keywords:** eosinophilic chronic rhinosinusitis, eosinophil, fibroblast, osteopontin, tissue remodeling

## Abstract

**Background:**

Eosinophilic chronic rhinosinusitis (ECRS) is a subtype of chronic rhinosinusitis (CRS) and is a refractory or intractable disease. However, a reliable clinical marker or an effective treatment strategy has not yet been established. ECRS is accompanied by excessive eosinophil infiltration and Th2 inflammatory response, which is closely related to tissue remodeling in the upper airways.

**Objectives:**

We sought to investigate the effect of eosinophils on tissue remodeling in ECRS. The purpose of this study was to identify the effects of eosinophils on the expression of pro-inflammatory mediators and extracellular matrix (ECM) in nasal fibroblasts and the key mediators that stimulate them.

**Methods:**

Butyric acid was used to differentiate EOL-1 cells into eosinophils. We co-cultured differentiated EOL-1 cells and fibroblasts to measure the expression of pro-inflammatory mediators and ECM in fibroblasts. Among the cytokines secreted from the differentiated EOL-1 cells, factors that induced tissue remodeling of fibroblasts were identified.

**Results:**

Treatment with butyric acid (BA) differentiated EOL-1 cells into eosinophils. Differentiated EOL-1 cells induced fibroblasts to produce pro-inflammatory mediators, IL-6 and IL-8, and tissue remodeling factor, VEGF. It also induced myofibroblast differentiation and overexpression of ECM components. Differentiated EOL-1 cells overexpressed osteopontin (OPN), and recombinant OPN increased the expression of IL-6, IL-8, VEGF, and ECM components in nasal fibroblast. OPN was overexpressed in the nasal tissue of patients with ECRS and was associated with the severity of CRS.

**Conclusions:**

Eosinophil-derived OPN stimulated nasal fibroblasts and contributed to inflammation and tissue remodeling in ECRS. Moreover, the expression level of OPN was proportional to the severity of ECRS. Therefore, OPN regulation is a potential treatment for ECRS.

## Introduction

Chronic rhinosinusitis (CRS) is a multifactorial inflammatory disease involving the nose and paranasal sinus. The phenotype of CRS is heterogeneous and is classified into CRS with nasal polyp (CRSwNP) and CRS without nasal polyp (CRSsNP) depending on the presence or absence of nasal polyp. However, the pathogenesis of the disease is difficult to determine from this classification. Hence, providing customized treatment becomes challenging as it is difficult to distinguish the immune response, disease progression, and histopathological differences in patients (1–3). Therefore, currently, the “inflammatory endotype” method that divides CRS based on T-helper cells, and its downstream mechanisms is used. This method classifies CRS into eosinophilic CRS (ECRS) and non-eosinophilic CRS (non-ECRS) (4).

ECRS is mainly observed in western countries. In ECRS, Type 2 inflammation is dominant along with tissue eosinophilia and is difficult to treat. Tissue eosinophilia is highly involved with recalcitrant nature of the disease and nasal polyp recurrence rate after endoscope sinus surgery. The tissues of patients with ECRS mainly show edema. ECRS is more severe than non-ECRS, with higher rates of recurrence. In contrast, non-ECRS is more prevalent in Asia. Glandular hyperplasia and dense collagen accumulation are observed among Asian patients (5, 6). Kim compared the patients in Asia from 1990-2000 and 2000-2010. The eosinophils per HPF in tissues increased by approximately 3-fold, from 6 to 18. The proportion of patients with ECRS also increased. He reported that these changes were related to the apparent westernization of Asian countries (7). Accordingly, the proportion of patients with ECRS has been on a rise globally, along with the rate of patients with refractory CRS, suggesting that the proportion of patients with high disease severity is increasing (8).

Eosinophils are one of the granular innate immune cells and play a key role in type 2 inflammation. Eosinophils differentiate into a mature form, mediated by IL-5, and infiltrate into the tissues, inducing type 2 inflammation (9). Tissue eosinophilia in the airway is associated with tissue remodeling and inflammation and is involved with the pathogenesis of local fibrosis, edema, allergic rhinosinusitis, asthma, and CRS (10, 11). Excessive eosinophil infiltration is observed in the subepithelial region of the nasal polyp tissue of ECRS patients, and extracellular matrix deposition is characteristically observed in the middle turbinate part where the nasal polyp form (6, 12).

EOL-1 is a human eosinophilic cell line and represents a useful *in vitro* model for eosinophil study. EOL-1 cell can be differentiated into eosinophil by butyric acid (BA). In previous studies, it was found that BA treatment of EOL-1 increased the expression of eosinophil markers such as CCR3, IL-5Ra, and eosinophil secondary granules (ECP, EDN) and differentiated them into eosinophil-like cells. It was also reported that eosinophil TLR receptors (TLR3, TLR4, TLR7, TLR8, and TLR9) were expressed in differentiated EOL-1 cells. In *in vitro* studies, primary eosinophils have disadvantages such as low survival rate and difficult long-term co-culture, so the EOL-1 cell line is often used as an alternative cell (13–15).

Fibroblasts are the primary cells to synthesize extracellular matrix (ECM), collagen, and stroma (16), and also contribute to inflammation. They are also involved in the maintenance of the tissue morphology (17). Fibroblasts are recognized for their limited role in wounded tissues. However, recent studies have indicated that fibroblasts are involved in the entire process of wound healing, right from inflammation to tissue remodeling. A close relationship between fibroblasts and inflammation has also been reported in many chronic inflammatory diseases involving continuous formation of micro-wounds *via* repeated insults to the tissue (18).

Therefore, in this study, we first aimed to evaluate the effects of eosinophils on tissue remodeling, and inflammatory properties of fibroblasts. Second, we tried to identify the key mediators involved in the eosinophil-mediated stimulation of fibroblasts. Finally, the effect of the eosinophil-derived mediator on the development and severity of CRS was confirmed.

## Materials and Methods

### Human Subjects

Uncinate process samples from the nasal cavity, and polyp tissues were obtained from 25 patients (7 males and 18 females). Normal uncinate process tissues were harvested during rhinoplastic surgery. Nasal polyp tissues were obtained from the region of the middle meatus at the beginning of the endoscopic surgical procedure of CRS patients. The CRS was diagnosed based on historical, endoscopic, and radiographic criteria and CT findings of sinuses according to the 2012 European position paper on rhinosinusitis and nasal polyps (EPOS) guidelines. All subjects were recruited from the Department of Otorhinolaryngology, Korea University Medical Center, Korea. All tissues were derived from patients with no signs of inflammation, allergies, asthma, or aspirin sensitivity. No patients had a history of consuming oral steroids, non-steroidal anti-inflammatory drugs, antihistamines, or antibiotics for at least past 2 months. Informed consent was collected according to the Declaration of Helsinki. This study was approved by the Korea University Medical Center Institutional Review Board, which also authorized the research. It was carried out in accordance with the guidelines of the Human Ethics Committee of the Korea University Guro Hospital (2020GR0308). Clinical characteristics of patients are summarized in **Table 1**.

**Table 1:** Clinical characteristics of patients (N=25).

Characteristic	UP (n= 3)	CRSsNP-UP (n= 5)	CRSwNP-UP (n= 6)	CRSwNP-NP (n= 11)
No. Women/men	2/1	1/4	1/5	3/8
Age, y, mean(SD)	31.7 (1.25)	46.6 (5.64)	45.4 (18.83)	46 (16.55)
Astma, no.	0	0	0	0
Aspirin sensitivity no.	0	0	0	0
Lund-Mackay CT score, mean (SD)	0.67 (0.47)	8 (2.94)	13.5 (7.95)	18 (4.93)

UP, uncinated process; NP, nasal polyp; CRSsNP, chronic rhinosinusitis without nasal polyp; CRSwNP, chronic rhinosinusitis with nasal polyp; SD, standard deviaon.

### Gene Set Enrichment Analysis (GSEA)

GSEA was performed to determine whether inflammation and ECM regulation are involved in CRS. GSEA software v4.1.0 was used to analyze the NGS data and GSE36830 data. Gene sets were analyzed using 10 hallmarks involving the inflammatory response and NABA ECM regulators. The number of permutations was set as 1000, the type was gene_set, and the chip platform was human_NCBI_Gene_ID_MSigDB.v7.4.chip.

### Cell Culture

#### Nasal Fibroblast Culture

Nasal fibroblasts were obtained from the inferior turbinates of patients who underwent rhinoplasty. Inferior turbinate tissues were subjected to enzymatic digestion with collagenase (500 U/mL; Sigma-Aldrich, St. Louis, MO), hyaluronidase (30 U/mL, Sigma), and DNase (10 U/mL, Sigma) to obtain fibroblasts. The cells were cultured in Dulbecco’s Modified Eagle Medium (DMEM) containing 10% heat-inactivated fetal bovine serum (FBS) (Invitrogen, Carlsbad, CA), 10,000 μg/mL streptomycin (Invitrogen), and 1% 10,000 U/mL penicillin (Sigma). The purity of the obtained nasal fibroblasts was confirmed by characteristic spindle-shaped cell morphology and flow cytometry. Fibroblasts were seeded with 7x10^5^ cells in a 100mm culture dish, and 3x10^5^ cells in a 60mm culture dish.

#### EOL-1 Culture

EOL-1 cells were purchased from Sigma and cultured in RPMI-1640 media supplemented with 10% heat-inactivated FBS (Invitrogen, Carlsbad, CA), 10,000 μg/mL streptomycin (Invitrogen), and 1% 10,000 U/mL penicillin (Sigma). EOL-1 were seeded with 1x10^6^ cells in a 100mm culture dish, and 5x10^5^ cells in a 60mm culture dish.

#### Co-Culture

EOL-1 cells were cultured in RPMI-1640 media containing 10% FBS and treated with 0.5 mM butyric acid (BA) to differentiate them into eosinophils. Differentiated cells were co-cultured with fibroblasts in the DMEM medium containing 10% FBS at a ratio of 1:2 and 1:4. In 60mm culture dish, fibroblasts were seeded with 3x10^5^ cells, and EOL-1 were seeded with 6x10^5^ and 1.2x10^6^, respectively. In addition, indirect culture was performed using a transwell. Differentiated EOL-1 cells were cultured in the upper chamber, and the fibroblasts were cultured in the lower chamber.

### Cell Viability Assay

#### Incucyte ^®^ Live Cell Analysis

To determine the cytotoxicity of butyric acid, IncuCyte ***^®^*** Cytotoxicity Assay (Essen BioScience Inc., Ann Arbor, MI) was performed. Briefly, EOL-1 cells were seeded into a 96-well plate at a density of 2 x 10^4^ cells per well. After seeding, the medium was replaced with butyric acid and treated with IncuCyte ***^®^*** Cytotox Green Reagent (Essen BioScience Inc.). The viability of the EOL-1 cells cultured at 37˚C in an atmosphere containing 5% CO2 and 95% humidity was measured at 72 h. Apoptotic EOL-1 cells were stained by IncuCyte ***^®^*** Cytotox Green Reagent and the viability of EOL-1 cells was evaluated by measuring green fluorescence through IncuCyte ***^®^*** software (Essen BioScience Inc.).

#### WST-1 Assay

To evaluate the cytotoxicity of butyric acid, WST-1 assay was performed. EOL-1 cells were seeded into a 96-well plate and 50 µL WST-1 reagent was added per well. The cells were incubated for 4 h and the results were obtained by measuring the absorbance using a spectrophotometer at 420-480 nM.

### Real-Time PCR

Total RNA was extracted using the Trizol reagent (Invitrogen). The cDNA was synthesized using the Maxime RT PreMix kit (Intron Biotechnology, Korea) according to the manufacturer’s protocol. Amplification reaction was performed with the following steps: an initial 2 min denaturation step at 94°C; 40 cycles of 94°C for 5 seconds, 60°C for 10 seconds, 72°C for 20 seconds. All reactions were performed in 20 μL volume. Real-time PCR was performed on the QuantStudio 3 system (Applied Biosystems, Foster City, CA) with 100 ng cDNA template, 400 nM of each primer, and 10 mL Power SYBR Green PCR Master Mix (Applied Biosystems) in 20 μL. Analysis of relative gene expression was conducted using the 2^-ΔΔCT^ method. Each experiment was repeated at least three times, and GAPDH was used as an internal control.

### Flow Cytometry Analysis

Flow cytometry was performed to confirm that EOL-1 cells differentiated into eosinophils. EOL-1 were seeded as 1x10^6^ cells in a 100mm culture dish and cells were treated with or without 0.5 mM BA and cells were harvested after 3-5 days. Cells were incubated with CCR3-PE (10 µL per 1x10^6^ cells, R&D Systems) and IL-5Ra-FITC (10 µL per 1x10^6^ cells, R&D Systems) conjugated antibodies for 30 minutes at 4°C and analyzed by FACS. All groups were analyzed with 10,000 cells. Flow cytometry analysis was performed using a Flow Cytometer Analyzer (LSR Fortessa X-20).

### Immunocytochemical Staining

To evaluate the protein expression and localization, immunocytochemical staining was performed. Before using the cell culture slides, they were coated with poly-L-lysine for 4 h, after which they were exposed to UV light for 6 h. Fibroblasts and EOL-1 cells were seeded on the poly-L-lysine coated slides. Subsequently, cells were fixed with 4% paraformaldehyde and permeabilized with 0.01% Triton X-100 in 1% bovine serum albumin for 10 minutes. Then, cells were blocked with 3% bovine serum albumin for 1 h. Fibroblasts were incubated with primary antibodies; anti-Fibronectin (1:200), anti-SMA (1:200), anti-collagen type I (1:200). Eol-1 were incubated with primary antibodies; anti-CCR3 (1:200), anti-IL-5Ra (1:200) and then incubated with secondary antibodies; anti-mouse Alexa 488 (Invitrogen) or anti-rabbit Alexa 555 (Invitrogen) secondary antibodies. Finally, cells were counterstained with 4’-6-diamidino-2-phenylindole for 10 min and stained cells were visualized using a confocal laser scanning microscope (LSM700, Zeiss, Oberkochen, Germany).

### Immunohistochemical Staining

Nasal polyp and uncinate process tissue sections were deparaffinized and rehydrated. Sections were stained with primary antibodies, anti-OPN (1:1,000, Abcam) and anti-IL5Ra (1:1,000, Abcam) overnight at 4°C. Subsequently, they were incubated with anti-mouse Alexa 488 (Invitrogen) or anti-rabbit Alexa 555 (Invitrogen) secondary antibodies or 3,3’- diaminobenzidine (DAB) substrate (Zytomed systems). Sections were counterstained with hematoxylin or 4’-6-diamidino-2-phenylindole (Sigma-Aldrich).

### ELISA

First, co-culture of fibroblasts and EOL-1 was performed. In 60mm culture dish, fibroblasts were seeded with 3x10^5^ cells, and EOL-1 were seeded with 6x10^5^ and 1.2x10^6^, respectively. After 3 days of co-culture, the levels of IL-6, IL-8, and VEGF were measured using ELISA (Biolegend, San Diego, CA, USA). In the second experiment, the expression levels of IL-6, IL-8 and VEGF were checked after 3 days of treatment with OPN (0.5-4ug/ml) in fibroblasts. The sample of ELISA was carried out using the supernatant from which cells were removed.

Briefly, each well was blocked with blocking buffer for 2 h and washed using a wash buffer. Antibodies against IL-6, IL-8, or VEGF were added to the media and incubated for 2 h. A substrate solution and stop solution were added sequentially, and the optical density of each well was determined within 30 minutes using a microplate reader (Bio-Rad).

### Cytokine Array

A cytokine and chemokine array (Proteome Profiler™ Human XL Cytokine Array Kit, R&D Systems, Minneapolis, MN, USA) was used to survey the changes in the expression levels of 102 cytokines and chemokines in EOL-1 cells after treatment with or without BA. Cell culture supernatant was collected, diluted, and incubated overnight with the contents of the array kit. The membrane was washed to remove unbound material followed by incubation with a cocktail of biotinylated detection antibodies. The membrane was incubated with streptavidin-horse radish peroxidase and chemiluminescent detection reagents, and a signal was generated at each capture spot corresponding to the amount of bound protein. The membrane was exposed to X-ray film for 10 minutes, and profiles of mean spot pixel density were analyzed using Quantity One software (Bio-Rad).

### *Ex Vivo* Organ Culture of Inferior Turbinate

Nasal inferior turbinate and polyp tissues were cut into 2 to 3 mm^3^ pieces, washed three times with phosphate-buffered saline, and cultured in DMEM supplemented with 2% FBS (Invitrogen) 1% 10,000 units/mL penicillin, and 10,000 μg/mL streptomycin (Invitrogen).

### Statistical Analysis

Results were obtained from at least three independent experiments. Statistical significance of differences between control and experimental data was analyzed using the unpaired *t-*test or one-way analysis of variance followed by Tukey’s test (GraphPad Prism, version 5, GraphPad Software, San Diego, CA). Significance was established at a 95% confidence level and P < 0.05 indicated statistical significance.

## Results

### Eosinophil Promotes Inflammation and ECM Production in Patients With ECRS

**Figure 1:**
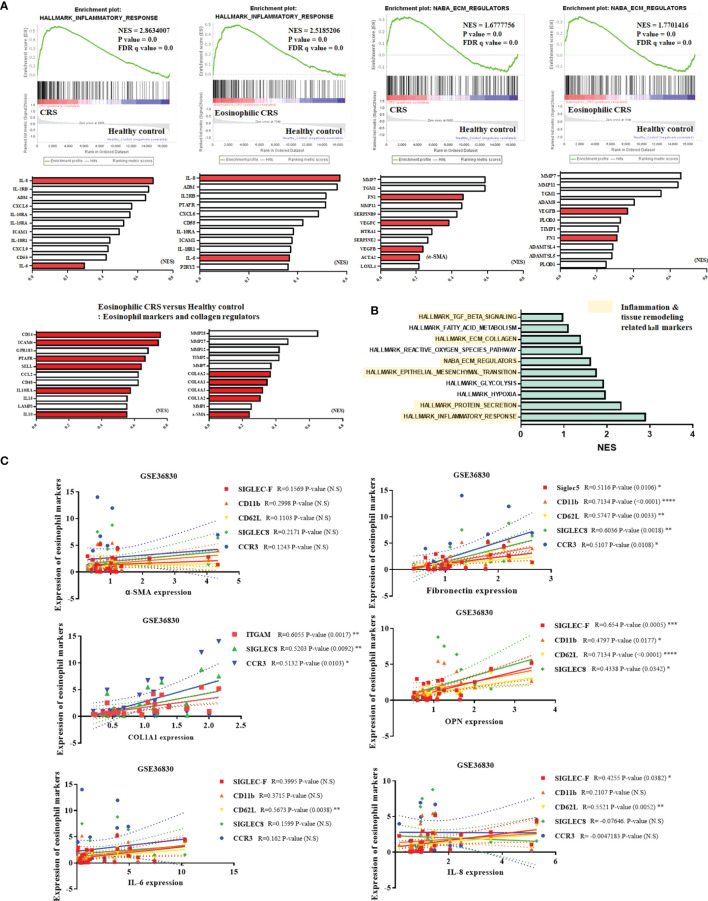
Correlation of eosinophils with inflammatory responses and production of ECM components in ECRS tissues. **(A)** GSEA comparing healthy control samples and samples derived from patients with CRS. The gene set used the inflammatory response and ECM regulator hallmarks. Pro-inflammatory mediators (IL-6, IL-8), extracellular matrix related factors (Fibronectin, *a*-SMA, collagen), and eosinophil markers are shown in red graphs. The graph shows the arrangement of genes based on NES scores. **(B)** Displaying a gene set with high NES scores obtained through GSEA. Hall markers related to inflammation and tissue remodeling are marked with yellow blocks. **(C)** Analysis of correlation between eosinophil markers and pro-inflammatory mediators, ECM components, and OPN (GSE36830 Data analysis). **(C)** was analyzed through the tissues of a total of 24 CRS patients. Data are analyzed with person correlation analysis. The significance values of the p-values of each data were expressed as 0.05 (*), 0.01 (**), 0.001 (***), and 0.0001 < (****).

We performed GSEA to determine the degree of inflammation and the amount of ECM produced in ECRS along with the effect of eosinophil on these aspects. The gene set database used Hallmark_inflammatory_response and NABA_ECM_Regulators. We observed that the correlation between inflammation and the expression of ECM regulators was higher in the CRS patient group than in the healthy control group, and a significant correlation was also found with the ECRS patient group. Among them, genes such as those encoding pro-inflammatory mediators IL-6 and IL-8 and ECM markers such as a-SMA and fibronectin showed a high normalized enrichment score (NES). (**Figure 1A**). When GSEA was performed using various genesets, high NES values ​​were derived for inflammation, tissue remodeling such as ECM and EMT, and protein secretion (**Figure 1B**). To examine whether eosinophils correlated with the expression of pro-inflammatory mediators and ECM components, correlation analysis was performed with the markers CD11b, CD62L, siglec-8, siglec-F, and CCR3. A partially positive correlation was confirmed (**Figure 1C**).

### Butyric Acid Induces Differentiation of EOL-1 Cells Into Eosinophils

**Figure 2:**
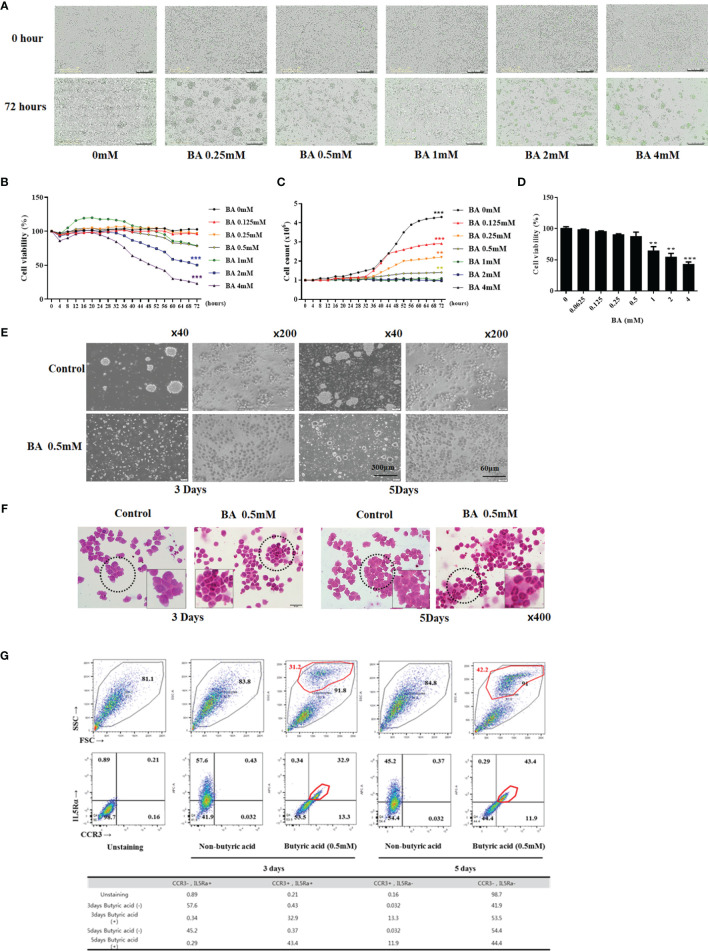
Butyric acid differentiates EOL-1 cells into eosinophils. **(A)** Live images (0 - 72 h) of EOL-1 cells treated with 0-4 mM BA. Cells emitting green fluorescence are dead cells. Scale bar = 300 µM. **(B, C)** Viability and proliferation graphs of cells treated with 0 - 4 mM BA (0 - 72 hours). **(D)** WST-1 assay. **(B-D)** All the results are presented as mean ± SD. Statistical significance was determined by one-way ANOVA test. Results were from at least three independent experiments. **p < 0.01 *vs*. BA 0mM; ***p < 0.001 *vs*. BA 0mM. **(E)** Phase-contrast images of EOL-1 cells and EOL-1 cells treated with BA at 0.5 mM. (x40, scale bar = 300 µm, x200, scale bar=60 µm) **(F)** H&E Staining. **(G)** Flow cytometry results of EOL-1 cells treated with 0.5 mM BA for 3 days and 5 days, respectively. Upper panel (X-axis=FSC, Y-axis=SSC). Bottom panel (X-axis=CCR3, Y-axis=IL-5RA staining).

We used BA to differentiate EOL-1 cells into eosinophils. Incucyte-based cytotoxicity assay and WST-1 assay were performed to confirm the effect of BA on the viability of EOL-1 cells. When the cells were treated with BA at a concentration of 0 – 0.25 mM, EOL-1 cells showed a survival rate of 96% to 100%, and approximately 78% when treated with 0.5-1 mM. In contrast, when treated with 2 mM and 4 mM concentrations, EOL-1 cells showed 50.3% and 23.05% survival rates, respectively (**Figure 2A, B**). When not stimulated with BA, EOL-1 cells showed a doubling time of approximately 16.74 h. Treatment with BA inhibited cell proliferation. Treatment with BA at a concentration of 0.5 mM showed a doubling time of approximately 51.42 h, and the proliferation of EOL-1 cells was reduced by 68% compared to EOL-1 cells cultured under normal conditions. Treatment with 4 mM BA completely inhibited the proliferation of EOL-1 cells (**Figure 2C**). In WST-1 assay, concentrations of BA over 1 mM exerted cytotoxicity (**Figure 2D**). To determine whether BA induced differentiation of EOL-1 cells into eosinophils, we examined the morphology and eosinophil markers in EOL-1 cells. EOL-1 cells were treated with 0.5 mM BA for 3 and 5 days and the cell morphology was confirmed by using an inverted microscope and H&E staining. EOL-1 cells, when not stimulated with BA, proliferated during the cell growth phase and formed colonies. However, the induction of differentiation in EOL-1 cells by BA decreased their ability to form colonies and proliferate (**Figure 2E**). H&E staining results showed that EOL-1 cells without BA stimulation were approximately 15-20 µm in size, present the form of a myeloblast and were circular or oval shaped. EOL-1 cells treated with BA exhibited a size of 9-17 µm and were slightly smaller than normal EOL-1 cells, with the two lobes of the nucleus being clearly visible (**Figure 2F**). CCR3 is a receptor for the C-C type chemokine and is highly expressed in eosinophils. IL-5Ra is a subunit of the IL-5 receptor. It primarily binds to IL-3, colony-stimulating factor-2, and IL-5, and affects the activation of eosinophils. We confirmed the expression of the eosinophil marker in untreated EOL-1 cells and EOL-1 cells treated with BA using flow cytometry. In EOL-1 cells without BA stimulation, approximately 40-50% of cells expressed IL-5Ra but did not express CCR3. In contrast, EOL-1 cells treated with BA for three days showed 32.9% of all cells, and 43.4% of EOL-1 cells treated for 5 days were double-positive CCR3 and IL-5Ra (**Figure 2G**).

### Differentiated EOL-1 Cells Up-Regulate the Expression of IL-6, IL-8, VEGF, and ECM Components in Nasal Fibroblasts

**Figure 3:**
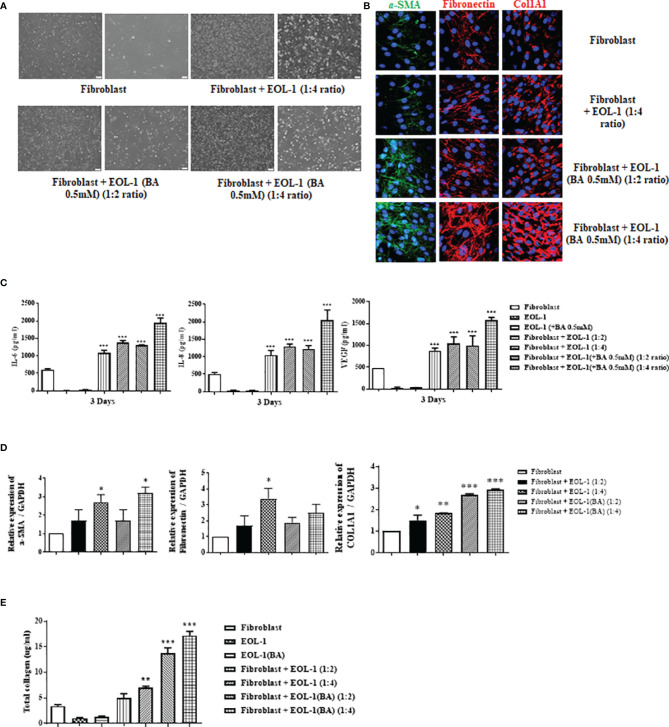
Differentiated EOL-1 cells induce the expression of IL-6, 8, and VEGF along with the production of ECM components in nasal fibroblasts. **(A)** Phase-contrast images obtained after the co-culture of fibroblasts and EOL-1 cells (scale bar=60 µM). **(B)** After co-culture of fibroblast and EOL-1 cells, expression of ECM components was confirmed in fibroblasts. Representative immunocytochemistry fluorescence images. (Green = α-SMA, Red = Fibronectin, collagen type 1, Blue = DAPI). **(C)** ELISA with supernatants obtained following co-culture. Evaluation of the IL-6, IL-8, and VEGF secretions. **(D)** After co-culture of fibroblasts and EOL-1 cells, RNA was extracted from fibroblasts and gene expressions of ECM components were evaluated using real-time PCR. **(E)** Sircol assay was performed using the supernatants. All the results are presented as mean ± SD. Statistical significance was determined by one-way ANOVA test. Results were from at least three independent experiments. *p < 0.05 *vs*. fibroblast; **p < 0.01 *vs*. fibroblast; ***p < 0.001 *vs*. fibroblast.

To confirm the effect of EOL-1 cells on fibroblasts, we co-cultured fibroblasts and EOL-1 cells at a ratio of 1:2 and 1:4 (**Figure 3A**). After 72 h of co-culture, EOL-1 cells were removed and the expression of tissue remodeling markers, namely, a-SMA, fibronectin, and collagen type I in fibroblasts was analyzed, followed by confirmation of the expression by immunohistochemistry. In fibroblasts co-cultured with differentiated EOL-1 cells, the expression of a-SMA, fibronectin, and collagen type I was significantly increased; moreover, the expression of ECM components was further upregulated in the group cultured with EOL-1 cells at a ratio of 1:4 (**Figure 3B**). Fibroblasts are traditionally recognized for their role in the production of the extracellular matrix. However, recent studies have indicated that fibroblasts produce pro-inflammatory mediators in addition to tissue remodeling factors. Hence, we examined the effect of the co-culture of EOL-1 cells and fibroblasts on the expression of pro-inflammatory mediators and VEGF. EOL-1 cells also upregulated the expression of pro-inflammatory mediators and VEGF in fibroblast. When fibroblasts were co-cultured with EOL-1 cells at a ratio of 1:2, 1:4, the secretion of IL-6, IL-8 and VEGF was increased. When co-culture was performed using differentiated EOL-1, higher production of IL-6, IL-8 and VEGF was observed (**Figure 3C**).

In co-culture conditions, RNA levels of ECM components (a-SMA, Fibronectin and collagen type I) of fibroblasts were partially elevated. a-SMA and collagen type 1 showed the greatest increase in RNA levels under co-culture conditions with differentiated EOL-1. However, Fibronectin did not show a significant increase in gene expression (**Figure 3D**). Total soluble collagen was measured using the Sircol assay. EOL-1 cells activated the fibroblast collagen production. Differentiated EOL-1 cells led to more collagen production compared to EOL-1 cells without differentiation (**Figure 3E**).

### Eosinophil-Fibroblast Contact Is Not Required to Induce the Expression of IL-6, IL-8, VEGF, and Total Collagen in Nasal Fibroblasts

**Figure 4:**
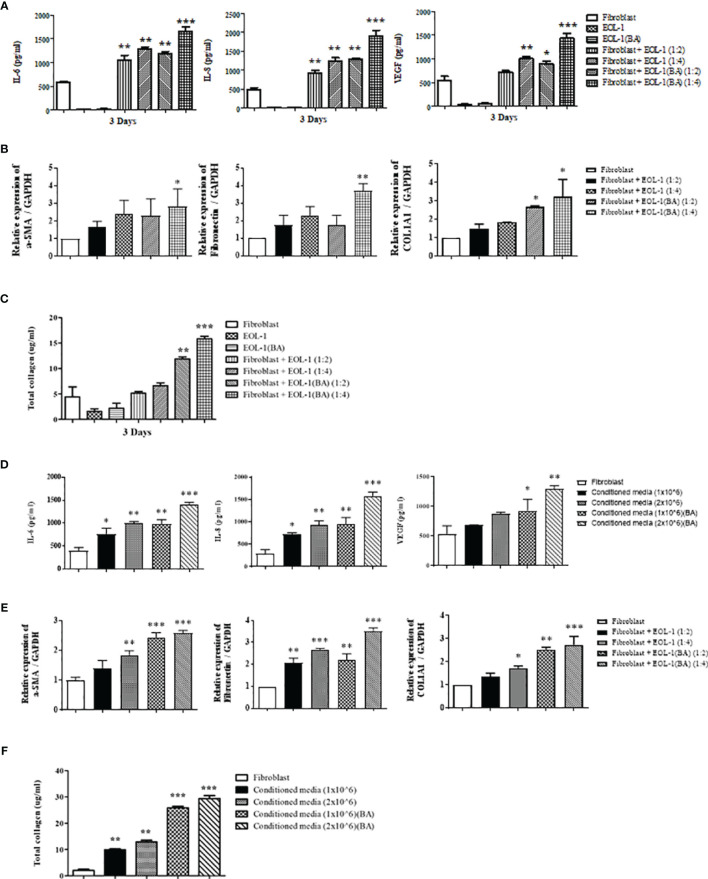
Eosinophil-fibroblast contact is not required for the induction of IL-6, IL-8, VEGF, and total collagen secretion by nasal fibroblasts. **(A)** After indirect co-culturing of fibroblasts and EOL-1 cells using a transwell, the supernatant was collected, and the secretion of IL-6, IL-8, and VEGF was measured by ELISA. **(B)** After indirect co-culturing of fibroblasts and EOL-1 cells using a transwell, the expression levels of the genes encoding for ECM component (a-SMA, fibronectin, collagen type 1) were confirmed in fibroblasts by real-time PCR. **(C)** The supernatant was examined for soluble collagen level using Sircol assay. **(D)** After the fibroblasts were treated with EOL-1 cell-conditioned media, the supernatant was collected, and the secretion of IL-6, IL-8, and VEGF was measured using ELISA. **(E)** Changes in the expression of genes encoding ECM component (a-SMA, fibronectin, collagen type 1) in fibroblasts treated with EOL-1-conditioned media were confirmed by real-time PCR. **(F)** The supernatant was examined for soluble collagen level using Sircol assay. All the results are presented as mean ± SD. Statistical significance was determined by one-way ANOVA test. Results were from at least three independent experiments. *p < 0.05 *vs*. fibroblast; **p < 0.01 *vs*. fibroblast; ***p < 0.001 *vs*. fibroblast.

**Figure 3** shows changes in the expression of IL-6, IL-8, VEGF, and ECM components in response to direct co-culture of EOL-1 cells and fibroblasts. However, we did not know which cells released those mediators. Hence, we physically separated EOL-1 cells and fibroblasts using transwell and evaluated whether the mediators secreted from EOL-1 cells affect the production of ECM components and cytokines in fibroblasts. After inserting the transwell into a 12-well plate, fibroblasts were cultured in a 12-well plate and EOL-1 cells were cultured in the transwell, respectively. At first, EOL-1 cells were treated with BA for 3 days before co-culturing with fibroblasts. Subsequently, differentiated EOL-1 cells were transferred in a transwell. IL-6, IL-8, and VEGF were mainly secreted from fibroblasts, not EOL-1 cells, and the secretion increased in fibroblasts co-cultured with differentiated EOL-1 cells (**Figure 4A**). EOL-1 cells also induced the expression of genes encoding ECM components. The differentiated EOL-1 cells further stimulated fibroblasts to increase the expression of ECM components (a-SMA, fibronectin, COL1A1) (**Figure 4B**). In addition, EOL-1 cells significantly increased the production of soluble collagen in fibroblasts (**Figure 4C**). Based on these results, we confirmed that EOL-1 cells stimulated fibroblast without direct interaction, leading to the production of inflammatory mediators, VEGF, and ECM components.

To confirm that the mediators secreted by EOL-1 cells play an important role in fibroblast stimulation, we used EOL-1 cell-conditioned media to treat the fibroblasts. After culturing EOL-1 cells with or without BA for 3 days, EOL-1 cells were removed by centrifugation, and the media was used for fibroblast culture. EOL-1 cell-conditioned media stimulated fibroblasts to increase the expression of IL-6, IL-8, and VEGF. When the EOL-1 cell ratio was higher and when differentiated EOL-1 cells were used, the expression of inflammatory mediators and VEGF was increased (**Figure 4D**). The expression of ECM components (a-SMA, fibronectin, and collagen type I) and soluble collagen showed similar results (**Figure 4E, F**). Therefore, we confirmed that cytokine and chemokine secreted from EOL-1 cells play an important role in fibroblast stimulation.

### Differentiated EOL-1 Cells Induce Osteopontin Expression

**Figure 5:**
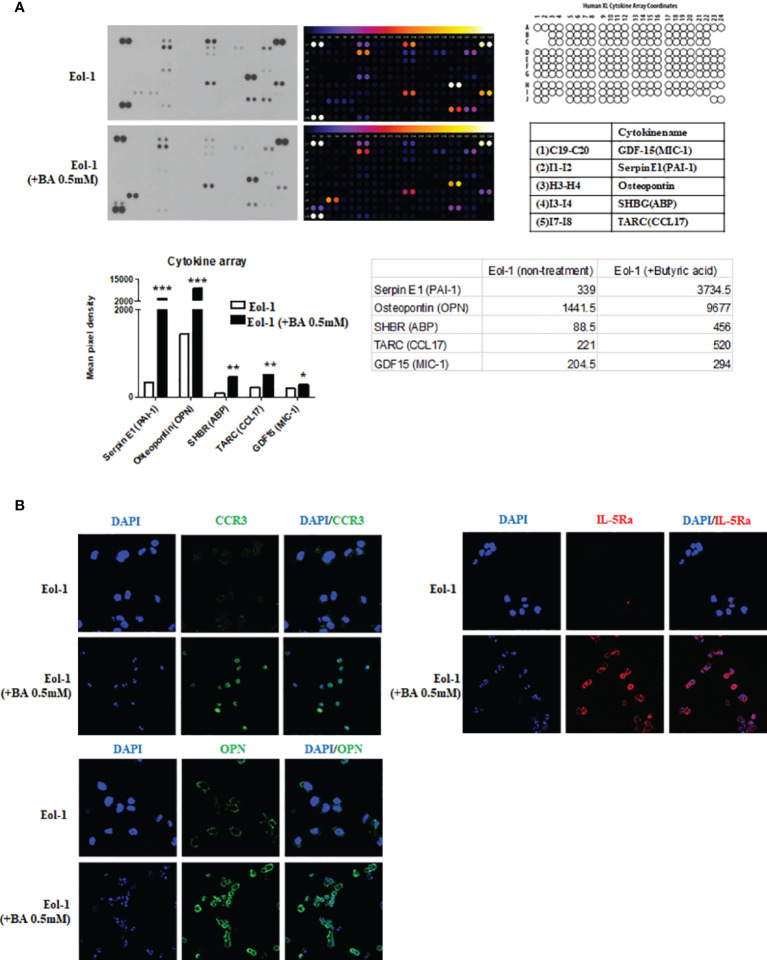
Differentiated EOL-1 cells induce the expression of osteopontin. **(A)** Analysis of cytokine expression in EOL-1 cells using the Human XL Cytokine Array. Supernatants of EOL-1 cells treated with or without 0.5 mM BA were collected and analyzed. Expression levels of 114 cytokine were analyzed, and five cytokines with a highly upregulated expressions are shown in the representative graphs. All the results are presented as mean ± SD. Statistical significance was determined by one-way ANOVA test. *p < 0.05 *vs*. target cytokine expression of EOL-1; **p < 0.01 *vs*. target cytokine expression of EOL-1; ***p < 0.001 *vs*. target cytokine expression of EOL-1. **(B)** Representative immunocytochemical images of EOL-1 with and without butyric acid (0.5 mM). (Green: CCR3, OPN, Red=IL-5Ra, Blue=DAPI).

We used a cytokine array to identify the key cytokine secreted from differentiated EOL-1 cells that alters the secretory capacity of fibroblasts After treatment of EOL-1 cells with 0.5 mM butyric acid for 3 days, the differences in cytokine expression compared to that from undifferentiated EOL-1 cells was analyzed using a human XL cytokine array kit. The expressions of serpin E1, osteopontin, and sex hormone binding receptor (SHBR) increased 11-fold, 6.7-fold, and 5.2-fold, respectively, in EOL-1 cells differentiated with butyric acid compared to those in undifferentiated EOL-1 cells (**Figure 5A**). Immunocytochemical analysis showed increased expression of CCR3 and IL-5Ra in differentiated EOL-1 cells, and overexpression of osteopontin (OPN) was also observed (**Figure 5B**).

### Osteopontin Induces the Expression of IL-6, IL-8, VEGF, and ECM Components in Nasal Fibroblasts

**Figure 6:**
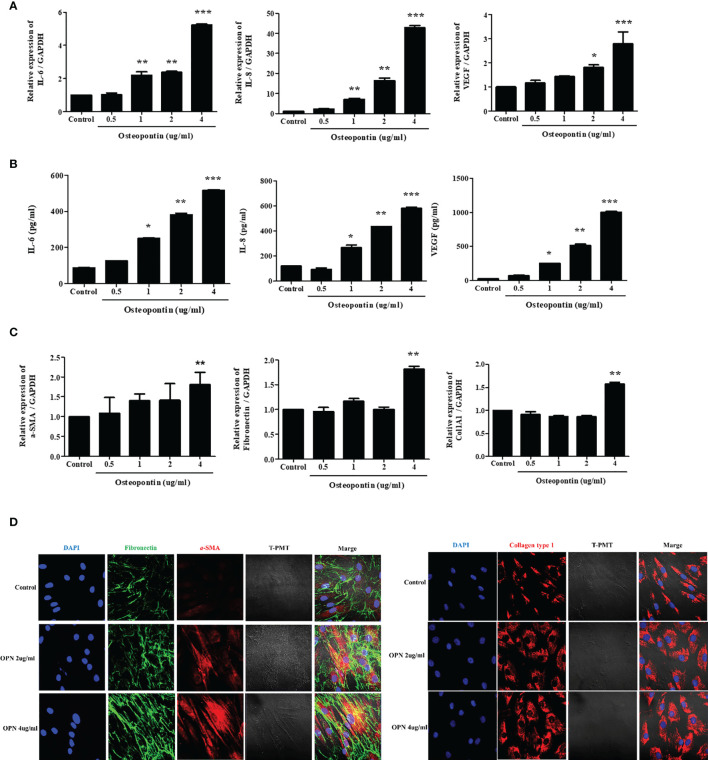
Osteopontin induces the expression of IL-6, IL-8, VEGF, and ECM components in nasal fibroblasts. **(A)** Fibroblasts were treated with OPN at concentrations ranging from 0.5 – 4 µg/mL, and the expression of IL-6, IL-8, and VEGF was measured using real-time PCR and **(B)** ELISA. **(C)** Expression of genes encoding ECM components under the same conditions using real-time PCR. **(D)** Fibroblasts were treated with 2 and 4 µg/mL OPN for 3 days, and the expression of ECM component proteins was measured by immunocytochemistry. All the results are presented as mean ± SD. Statistical significance was determined by one-way ANOVA test. Results were from at least three independent experiments. *p < 0.05 *vs*. control; **p < 0.01 *vs*. control; ***p < 0.001 *vs*. control. **(D)** Representative immunocytochemistry images (x400). Left panel (Blue=DAPI, Green=Fibronectin, Red=a-SMA), right panel (Blue=DAPI, Red=Collagen type I).

To evaluate the effects of OPN on nasal fibroblasts, we directly treated fibroblasts with osteopontin, a cytokine overexpressed in differentiated EOL-1 cells. Fibroblasts were treated with osteopontin at concentrations ranging from 0.5-4 µg/mL for 48 h and 72 h, respectively, and RNA and protein levels were measured using real-time PCR and ELISA. Recombinant OPN significantly increased the expression of IL-6, IL-8, and VEGF in fibroblasts in a dose-dependent manner (**Figure 6A, B**). RNA levels of a-SMA were significantly increased only upon treatment with OPN at a concentration of 4 µg/mL or more. The expressions of fibronectin and collagen type I were also elevated upon treatment with OPN at 4 µg/mL (**Figure 6C**). Immunocytochemical analysis showed that OPN significantly increased the protein expression of ECM components (a-SMA, fibronectin, collagen type I) in fibroblasts. All the ECM components were expressed in the fibroblast cytoplasm, with the most significant increase observed upon treatment with 4 µg/mL OPN (**Figure 6D**).

### Osteopontin Expression in Nasal Tissues

**Figure 7:**
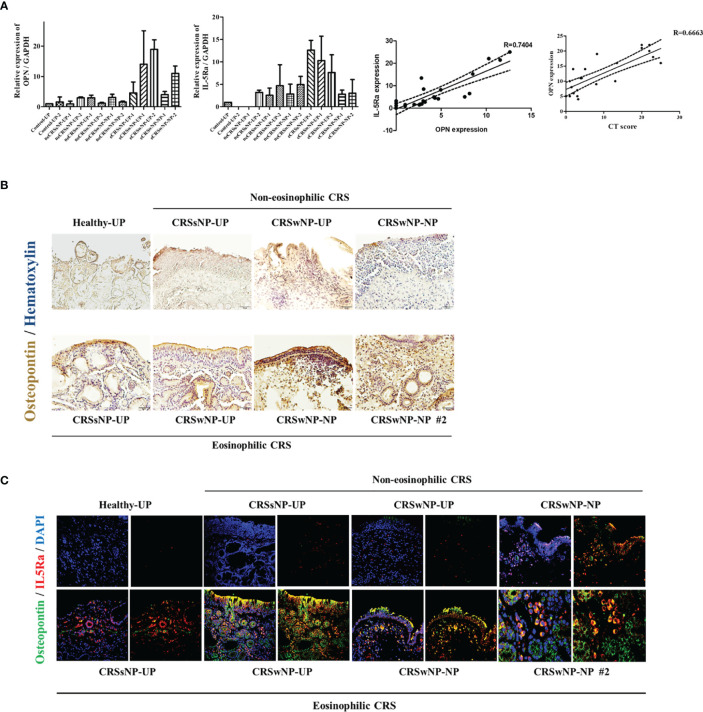
Expression of osteopontin in nasal tissues. Gene and protein expression were analyzed using uncinate process and nasal polyp tissues of a total of 25 patients in the normal group and CRS patient group (7 males and 18 females). **(A)** Expression of OPN and IL-5Ra encoding genes in nasal tissues (UP and polyp) of normal group and CRS patient group. Representative graph showing the correlation between the expression levels of OPN and IL-5Ra. A representative graph of the correlation between CT Score and OPN expression. **(B)** Representative immunohistochemistry image showing the expression of OPN in the nasal tissues (UP and polyp) of the normal group and CRS patient group (x200) (Brown=OPN, Blue=Hematoxylin) and **(C)** immunohistochemistry fluorescence image (Blue=DAPI, Green=OPN, Red=IL-5Ra) (x200).

To evaluate the effect of OPN on CRS, we examined the expression of OPN in the nasal tissues. The expression of OPN was higher in CRS tissues than in healthy control tissues. Especially, a significant increase was observed in the ECRS. IL-5Ra, an eosinophil marker, was highly expressed in the ECRS tissue, and the expression of OPN and IL-5Ra showed a positive correlation. The expression level of OPN showed a significant correlation with the severity of CRS (CT score) (**Figure 7A**). IHC was performed to examine the expression of OPN in tissues. OPN was primarily overexpressed on the surface of nasal epithelial cells, endothelial cells in blood vessels, and eosinophils. The expression level was increased in ECRS tissue (**Figure 7B**). Results of IHC showed high expression of OPN in ECRS (Green), and co-staining was observed in IL-5Ra (Red) + cells; eosinophils (**Figure 7C**).

## Discussion

In this study, we investigated the effect of eosinophils on the inflammation and tissue remodeling of ECRS, and particularly, the mechanism by which eosinophils induce the secretion of pro-inflammatory mediators and ECM overproduction in nasal fibroblasts. Activated eosinophils stimulated nasal fibroblasts *via* hypersecretion of OPN, which differentiated fibroblasts into myofibroblasts with subsequent overproduction of ECM. In addition, the inflammatory response in the tissue was sustained and strengthened *via* the secretion of IL-6 and IL-8 by fibroblasts. The expression of OPN was high in patients with ECRS, and there was a significant correlation between the expression of OPN and the severity of ECRS.

Typically, tissue remodeling and inflammatory responses are initiated to localize and eliminate the invasion of injurious agents and rebuild the tissue normally in the presence of harmful stimuli, such as invasion by pathogens (19, 20). However, in chronic inflammatory diseases such as CRS, excessive tissue remodeling occurs as a result of a persistent inflammatory reaction, leading to the onset of processes such as collagen deposition, fibrosis, edema, thickening of basement membrane, and goblet cell hyperplasia (2, 21). Hence, there is a strong correlation between inflammatory response and tissue remodeling. The tissues of patients with ECRS mainly show edema patterns with higher severity than patients with non-ECRS. Moreover, ECRS demonstrates higher rates of recurrence (6, 22).

Eosinophils are granulocyte leukocytes that play a key role in type 2 inflammation and are involved in cell-mediated immunity by penetrating tissues during parasitic infections or allergic diseases (23). In the respiratory tract, eosinophils play an important role in several chronic inflammatory diseases, including asthma, allergic rhinosinusitis, and chronic rhinosinusitis (24). Classically, the examination of the role of eosinophils in such diseases has been focused on the inflammatory response (23). However, recent studies have shown that the increase of eosinophils in the airway is also associated with tissue remodeling occurring *via* interaction with other cells. These eosinophils are also involved in regulating other cells around them (25). Eosinophils induce the epithelial-to-mesenchymal transition in bronchial cells, leading to remodeling of the epithelium (15, 26). Eosinophils control migration and infiltration of immune cells by regulating the surface marker expression in endothelial cells (27, 28). The fibroblasts are the main cells to synthesize collagen and contribute to tissue regeneration (19, 20). Moreover, fibroblasts not only play a role in tissue organization, but also contribute to the immune response *via* cytokine secretion (18, 29). As for the relationship between eosinophils with fibroblasts, eosinophils stimulate fibroblasts to increase the production of extracellular matrix and induce tissue fibrogenesis (30).

The interaction of heterogeneous and homogeneous cells is essential for biological function. *Via* interaction among these cells, the proliferation and characteristics of cells can be modulated. Physiologically, cells can affect other cells through physical contact or *via* secretory factors; these interactions can be bidirectional or multidimensional (31). The co-culture method is one of the experimental methods to identify such interactions, signaling mechanisms, and synergies between cells that cannot be observed by the monoculture method. It is used to identify individual and collective effects of physical contact and soluble factors that act *via* paracrine signals. In addition, in co-culture system, cell-cell contact can be controlled by establishing a physical barrier, so that it is possible to observe the effects of the secretory factors alone (32). Therefore, we used a direct co-culture system as well as an indirect co-culture system in which eosinophil and fibroblast were physically separated by a transwell. The interactions between fibroblasts and eosinophils, in both direct, or indirect culture, significantly increased the expression of proinflammatory cytokines (IL-6 and IL-8), angiogenic factor (VEGF), and ECM markers (alpha-SMA, fibronectin, collagen) compared to those observed in fibroblasts and EOL-1 cells cultured separately. Even culture media in which the EOL-1 cells were cultured could exert an equivalent stimulatory effect on fibroblasts. Hence, we inferred that the mediators secreted from EOL-1 cells can stimulate or regulate the fibroblasts.

EOL-1 is a human eosinophilic leukemia cell line, and the cells exhibit cytological features of myeloblasts under normal conditions. The size of the EOL-1 cells is larger than that of the eosinophils with a single nucleus. The cells can functionally differentiate into eosinophils in response to certain stimuli. Butyric acid (BA), a straight-chain fatty acid, can differentiate EOL-1 cells into mature eosinophil-like cells (33). Using flow cytometry, we confirmed that EOL-1 cells differentiated into eosinophils in response to BA treatment; CCR3 and IL-5R were used as markers to confirm the differentiation of EOL-1 cells into eosinophils. CCR3 is a receptor for C-C type chemokines, including eotaxin, eotaxin-3, monocyte chemoattractant protein-3 (MCP-3), and MCP-4, while IL-5Ra is an interleukin 5 specific subunit of a heterodimeric cytokine receptor. Both CCR3 and IL-5Ra are highly expressed in eosinophils (34).

We used a cytokine array to identify the components of EOL-1 cells that mediated cytokine secretion and ECM production by fibroblasts. EOL-1 cells differentiated with butyric acid showed differential cytokine expression pattern compared to EOL-1 cells cultured under normal conditions. In particular, the expression of OPN, Plasminogen activator inhibitor-1 (PAI-1), and sex hormone-binding receptor (SHBR) in differentiated EOL-1 cells increased 11-fold, 6.7-fold, and 5.2-fold, respectively, and the expressions of thymus and activation-regulated chemokine (TARC) and macrophage inhibitory cytokine 1 (MIC-1) were also slightly elevated. Among them, OPN showed stimulatory effects on fibroblasts comparable to those observed with co-culture experiments. OPN, also known as T lymphocyte activation 1 and secreted phosphoprotein 1, is a highly negatively charged ECM protein. It was discovered in the bone as an ECM protein (35). However, by binding to several integrin receptors such as α4β1, α9β1, and α9β4 expressed in leukocytes, OPN can affect cell migration and survival, and regulate the immune system (36). OPN is expressed in most immune cells such as eosinophils, neutrophils, dendritic cells, T-cells, and B-cells and is associated with the pathogenesis of inflammatory diseases (37). Furthermore, OPN has chemotactic properties and can recruit immune cells to the inflammatory sites and is also involved in cell attachment and wound healing processes. Earlier, it was known to mainly induce Th1 cytokine expression to contribute to tissue fibrosis, such as idiopathic pulmonary fibrosis (38). However, recent studies have shown that it is also involved in Th2-linked airway inflammatory diseases (39, 40). We examined whether the expression of OPN was of significance in the tissues of CRS patients.

OPN expression was increased in both CRSsNP and CRSwNP patients, and among them, the eosinophilic type of CRSwNP was most frequently expressed in the nasal polyp tissues of patients. The protein levels of OPN were significantly higher in eosinophilic NP tissues than in the non‐eosinophilic NP tissues. In addition, IHC double staining results confirmed that the eosinophils in the eosinophilic NP tissue were OPN+ cells.

Eosinophilic CRS is a relatively dominant type of eosinophil in the infiltrated immune cells in mucosa tissue. In fact, they show higher levels of inflammation and higher severity than non-eosinophilic ones. Therefore, the probability of recurrence after surgery is high and the prognosis is poor. Inflammatory response of nasal tissue caused by foreign antigen increases vascular permeability in the tissue and increases the residual eosinophil in the tissue. In this study, it was confirmed that these EOL-1 can secrete OPN. OPN has a chemotaxis effect that can move eosinophils into tissues, so it may accelerate tissue inflammation and tissue eosinophilia through positive feedback (6). The ability of OPN to induce tissue eosinophilia and upregulate the production of factors related to tissue remodeling may ultimately be closely related to tissue remodeling and severity of CRSwNP. In our results, the expression of OPN was higher in the uncinate process tissue than in the nasal polyp tissue of CRSwNP, and the number of infiltrated eosinophils was higher. Based on these results, OPN may act as a trigger to further accelerate the inflammation of CRSwNP in the early stage and to induce the formation of nasal polyp through the induction of irreversible tissue remodeling in the nasal cavity We plan to further investigate the effect of OPN in an eosinophilic mouse model made with Staphylococcal enterotoxin B (SEB) and OVA. Furthermore, we plan to conduct a study on whether tissue remodeling and nasal polyp formation are inhibited when SEB/OVA is treated in the OPN knockout mouse model, unlike the chronic rhinosinusitis mouse model.

Based on the finding that eosinophilic markers correlated with tissue remodeling markers in CRS patients, we investigated the effects of eosinophils on the fibroblasts of the upper airway along with identifying the factors secreted from eosinophils involved in this process. We confirmed that EOL-1 cells differentiated by BA increased the expression of mediators related to inflammation and tissue remodeling under three different co-culture methods. To identify the factors secreted by EOL-1 cells that stimulated the fibroblast to produce pro-inflammatory cytokines and mediated their differentiation into the active form, we used a cytokine array. Among the cytokines released from EOL-1 cells activated by BA, treatment with OPN alone showed similar effects on fibroblasts compared to the effects observed in co-culture experiments. Moreover, the expression level of OPN in tissues from CRS patients correlated with the level of eosinophil markers. Taking these results together, the level of OPN expression in tissues can be used as a diagnostic marker for ECRS. In addition, regulation of OPN expression could be a potential therapeutic strategy for ECRS.

## Data Availability Statement

The original contributions presented in the study are included in the article/supplementary material. Further inquiries can be directed to the corresponding author.

## Ethics Statement

This study was approved by the Korea University Medical Center Institutional Review Board, which also authorized the research. It was carried out in accordance with the guidelines of the Human Ethics Committee of the Korea University Guro Hospital (2020GR0308). The patients/participants provided their written informed consent to participate in this study.

## Author Contributions

H-WY conceived the study, designed and performed the experiments, analyzed the data, and wrote the manuscript. J-HP, M-S, J-MS, and DK confirmed the data and discussed this study. I-HP supervised the research and reviewed the manuscript. All authors reviewed the manuscript. All authors contributed to the article and approved the submitted version.

## Funding

This research was supported by a Korea University Grant (K1906871, K2117311).

## Conflict of Interest

The authors declare that the research was conducted in the absence of any commercial or financial relationships that could be construed as a potential conflict of interest.

## Publisher’s Note

All claims expressed in this article are solely those of the authors and do not necessarily represent those of their affiliated organizations, or those of the publisher, the editors and the reviewers. Any product that may be evaluated in this article, or claim that may be made by its manufacturer, is not guaranteed or endorsed by the publisher.
